# Long-Term Shedding of Influenza Virus, Parainfluenza Virus, Respiratory Syncytial Virus and Nosocomial Epidemiology in Patients with Hematological Disorders

**DOI:** 10.1371/journal.pone.0148258

**Published:** 2016-02-11

**Authors:** Nicola Lehners, Julia Tabatabai, Christiane Prifert, Marianne Wedde, Joe Puthenparambil, Benedikt Weissbrich, Barbara Biere, Brunhilde Schweiger, Gerlinde Egerer, Paul Schnitzler

**Affiliations:** 1 Department of Internal Medicine V, University of Heidelberg, Heidelberg, Germany; 2 Department of Infectious Diseases, Virology, University of Heidelberg, Heidelberg, Germany; 3 Institute of Virology and Immunobiology, University of Würzburg, Würzburg, Germany; 4 National Reference Centre for Influenza, Robert Koch Institute, Berlin, Germany; University of North Carolina at Chapel Hill, UNITED STATES

## Abstract

Respiratory viruses are a cause of upper respiratory tract infections (URTI), but can be associated with severe lower respiratory tract infections (LRTI) in immunocompromised patients. The objective of this study was to investigate the genetic variability of influenza virus, parainfluenza virus and respiratory syncytial virus (RSV) and the duration of viral shedding in hematological patients. Nasopharyngeal swabs from hematological patients were screened for influenza, parainfluenza and RSV on admission as well as on development of respiratory symptoms. Consecutive swabs were collected until viral clearance. Out of 672 tested patients, a total of 111 patients (17%) were infected with one of the investigated viral agents: 40 with influenza, 13 with parainfluenza and 64 with RSV; six patients had influenza/RSV or parainfluenza/RSV co-infections. The majority of infected patients (n = 75/111) underwent stem cell transplantation (42 autologous, 48 allogeneic, 15 autologous and allogeneic). LRTI was observed in 48 patients, of whom 15 patients developed severe LRTI, and 13 patients with respiratory tract infection died. Phylogenetic analysis revealed a variety of influenza A(H1N1)pdm09, A(H3N2), influenza B, parainfluenza 3 and RSV A, B viruses. RSV A was detected in 54 patients, RSV B in ten patients. The newly emerging RSV A genotype ON1 predominated in the study cohort and was found in 48 (75%) of 64 RSV-infected patients. Furthermore, two distinct clusters were detected for RSV A genotype ON1, identical RSV G gene sequences in these patients are consistent with nosocomial transmission. Long-term viral shedding for more than 30 days was significantly associated with prior allogeneic transplantation (p = 0.01) and was most pronounced in patients with RSV infection (n = 16) with a median duration of viral shedding for 80 days (range 35–334 days). Long-term shedding of respiratory viruses might be a catalyzer of nosocomial transmission and must be considered for efficient infection control in immunocompromised patients.

## Introduction

In winter 2012/2013, the number of viral respiratory tract infections in Germany was the highest observed during the past decade. Infections with respiratory viruses are a common cause of usually mild respiratory illness in all age groups. Immunosuppressed adults and elderly persons with underlying chronic conditions, however, are at increased risk for a severe course of disease [1–4]. In hematopoietic stem cell recipients, respiratory viruses cause higher rates of lower respiratory tract disease and are associated with a higher mortality rate [5–9]. For patients with hematological disorders presenting with respiratory symptoms, a screening for influenza virus, parainfluenza virus and respiratory syncytial virus (RSV) is recommended [10, 11].

Although a vaccine against seasonal and pandemic influenza is available, vaccines against parainfluenza and RSV are still under development [6, 12]. However, the effect of vaccination in immunosuppressed patients is limited. As the major pathogen causing severe lower respiratory tract disease in immunocompromised adults, RSV is of high priority for vaccine development. RSV infections only partially induce protective immunity, and repeated infections occur in childhood and throughout life [13]. Strain variation in respiratory viruses is thought to contribute to their ability to cause frequent reinfections [14]. The attachment protein of RSV is able to accommodate changes with the emergence of new variants. Sequencing of hypervariable gene regions has been widely used to further subdivide parainfluenza and RSV into genotypes and facilitate differentiation between virus isolates. Influenza viruses are highly variable and characterized by a continuous genetic and antigenic drift. Accumulation of mutations especially in the antigenic sites of the hemagglutinin is the cause of the emergence of new drift variants and the co-circulation of different groups and lineages.

Viral shedding studies provide fundamental information about the natural course of respiratory virus infections, related clinical illness and the implementation of effective prevention strategies. Influenza is generally a self-limiting infection with systemic and respiratory symptoms, usually resolving within 3 to 6 days in most patients. Viral clearance in the respiratory tract usually occurs after 3 to 5 days [15]. However, in immunocompromised patients respiratory viruses tend to persist longer due to a constrained immune response and therefore also spread more easily into the lower respiratory tract. Prolonged influenza and RSV viral shedding has been previously described in immunocompromised patients [16–18] and similar results have been observed for rhinovirus and coronavirus [19]. However, there is only limited information regarding the molecular epidemiology of respiratory viruses in immunocompromised adults combined with the prevalence, duration and clinical impact of viral shedding.

In our study, we retrospectively investigated patients with respiratory tract infection in the hematology and transplant unit of the University Hospital Heidelberg between December 2012 and May 2013. We performed molecular characterization of influenza virus, parainfluenza virus and RSV investigating their genetic diversity and patterns of co-circulating subtypes and genotypes. Furthermore, we assessed the prevalence, duration and clinical impact of prolonged viral shedding in immunocompromised adults.

## Materials and Methods

### Patients and infection control

The Heidelberg University Hospital is a tertiary referral center. The department of hematology comprises four inpatient wards for adult patients—two wards for normal and high-dose chemotherapy, one intermediate care unit and one transplant unit—as well as several outpatient clinics and a day hospital where chemotherapy on an outpatient basis is administered. Most of the patients treated suffer from malignant lymphoma, multiple myeloma, and acute leukemia. Each year about 200–250 autologous and 100–120 allogeneic transplantations are performed. The majority of allogeneic transplant patients receive peripheral blood stem cells after a reduced conditioning regimen.

As part of the standard operation procedures and in order to inform for infection control measures, from autumn to spring all hematological patients are tested on development of respiratory symptoms for infection with influenza virus, parainfluenza virus and RSV (standard screening); during the summer months only patients with very severe or not otherwise explicable symptoms are tested due to the lower prevalence of respiratory virus infections in that season. With significantly increasing numbers of respiratory infections, standard screening was escalated in February 2013 and subsequently all patients were screened for infection with influenza virus, parainfluenza virus and RSV on admission to one of the wards regardless of respiratory symptoms as well as tested again once respiratory symptoms developed (intensified screening). Additional screening for other community acquired viruses such as rhino-, adeno- or coronavirus was not performed. All patients with proven viral infection were retested until clearance of virus. Several patients were lost to follow-up regarding their viral shedding. Furthermore, symptomatic patients were isolated while awaiting the laboratory result. Infected patients were isolated in single rooms or isolated in cohorts.

The samples were processed within two hours and afterwards kept frozen at -20°C. Analysis was performed with nasopharyngeal samples via reverse transcription polymerase chain reaction (RT-PCR). A case of respiratory tract infection was based on a laboratory-confirmed infection with influenza virus, parainfluenza virus or RSV presenting with or without respiratory symptoms. URTI was defined as presence of respiratory symptoms (e.g. coughing, sneezing) without signs of lower respiratory tract disease, LRTI was defined as presence of respiratory symptoms plus radiographic (chest X-ray or chest CT scan) signs of lower respiratory tract disease, severe LRTI was defined as LRTI plus requirement of treatment on the intensive care unit or death. Nosocomial infections were defined as virus detection on day seven or later after admission to the ward, the remainders were defined as community-acquired infection. However, some of the community-acquired cases had been previously treated at the day hospital. Patient records and information were anonymized and de-identified prior to analysis. Ethical approval was obtained from the Ethical Committee of the University of Heidelberg.

### Sample collection

For the purpose of this study, nasopharyngeal swabs of all inpatients and outpatients who were screened for the respiratory viruses influenza, parainfluenza and RSV during the winter season 2012/2013 were retrospectively analysed. For some of the LRTI patients, additional bronchoalveolar lavage (BAL) samples were available. Readily available medical records were retrospectively reviewed from all patients to obtain basic characteristics, clinical and laboratory data.

### Reverse transcription polymerase chain reaction (RT-PCR)

RNA was extracted from respiratory specimens using the QIAamp^®^ viral RNA mini kit (Qiagen, Hilden, Germany) according to the manufacturer’s protocol. Reverse transcription, amplification and detection of viral RNA was performed with the RealStar^®^ Influenza, Parainfluenza and RSV real-time RT-PCR kits (altona Diagnostics, Hamburg, Germany) on a LightCycler^®^ 480 instrument II (Roche, Mannheim, Germany) according to the manufacturer's instructions. These assays distinguished influenza A, B, H1N1, RSV A, and RSV B. The RealStar Parainfluenza RT-PCR kit cannot differentiate between parainfluenza virus types 1 and 3 and between types 2 and 4. Therefore, the FTD Respiratory pathogens 21 kit (Fast Track Diagnostics, Luxembourg) was used for typing of samples positive for parainfluenza virus RNA.

### Sequencing and phylogenetic analysis

Extracted RNA was reverse transcribed using random hexamer primers. Subsequently, influenza virus hemagglutinin (HA) gene, parainfluenza virus 3 F (fusion) gene and the second variable region of the RSV G (attachment) gene were amplified from cDNA. Primer sequences for amplification of influenza virus HA genes are available on request. For parainfluenza virus 3, primers PIV3-4885s (5’- AAAGAGGTCAACACCAACAACT-3’) and PIV3-5285a (5’- TGTATTGCTTGATCTGTTGGTC- 3’) were used. RSV primers were described previously [20]. Resulting PCR products were sequenced completely in both directions using Big Dye terminator chemistry version 1.1 and the instrument Prism 3130xl (Applied Biosystems, Darmstadt, Germany). Overlapping sequences were assembled using the SEQMAN II software of the Lasergene package (DNAstar, Madison, USA). Multiple alignments from influenza virus, parainfluenza virus and RSV nucleotide sequences were carried out with the MEGA software version 5.05. A phylogenetic tree was constructed in MEGA using the maximum-likelihood method and the Tamura-Nei algorithm for influenza virus phylogenetic analysis MEGA version 5.2, the neighbor joining method and the Kimura 2-parameter were used [21, 22]. Representative reference sequences for influenza, parainfluenza and RSV genotypes were obtained from GenBank (http://www.ncbi.nlm.nih.gov), GISAID (global initiative on sharing all influenza data, www.gisaid.org) and sequences representing influenza viruses circulating in Germany were provided by the National Reference Centre for Influenza and included in the phylogenetic analysis. The statistical significance of the tree topology was assessed by bootstrapping with 1000 replicates. Nucleotide sequences retrieved in this study were deposited in GenBank under accession numbers [KJ938237-KJ938299].

### Viral shedding

Follow-up of shedding of RSV-infected patients was conducted on wards as well as in the outpatient facility. Analysis of the duration of shedding was performed among all cases with samples available from at least two consecutive weeks. Total duration of viral shedding for a given infection was calculated from the first positive to the final positive sample, allowing no more than four weeks or one negative sample between any two consecutive positive samples. A previously infected patient was considered to have cleared the infection when he/she was asymptomatic and had two nasopharyngeal samples taken a week apart that tested negative

### Statistical analysis

We used Stata version 11 (Stata Corp., College Station, TX, USA) for all statistical analyses. Fisher’s exact test was applied for univariate analyses, logistic regression models were used for multivariate analyses.

## Results

### Epidemiology and laboratory results

**Fig 1:**
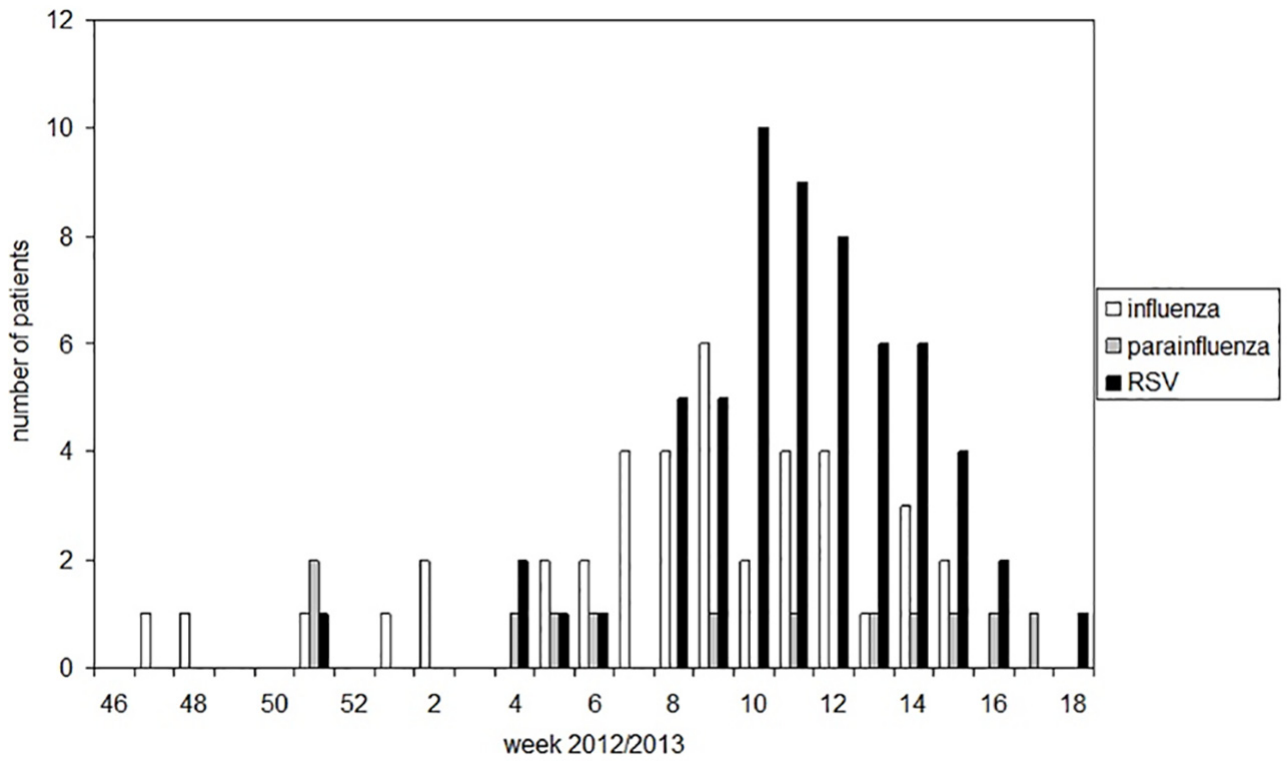
Cases of influenza virus, parainfluenza virus and RSV infections in the hematology unit during the winter of 2012/2013 by week of laboratory confirmation.

In November and December 2012, a few sporadic cases of patients with respiratory virus infection were detected by PCR. Escalated screening of all patients to be admitted was performed starting in week 8 to week 16 in 2013, when a significant increase of infected patients was observed. The total number of patients admitted for any reason to one of the four hematologic wards was 421 (148 female, 273 male) from November 2012 to January 2013, and 341 (123 female, 218 male) from February to April 2013. Between November 2012 and April 2013, a total of 672 patients with hematological disorders were tested for presence of influenza virus, parainfluenza virus or RSV, 111 patients were found to be infected, 40 patients were with influenza virus, 13 patients with parainfluenza virus and 64 patients with RSV, six patients had co-infections (influenza virus/RSV: n = 5; parainfluenza virus/RSV: n = 1). According to the case definition, 30 of 40 (75%) influenza infections, 11 of 13 (84%) parainfluenza infections and 36 of 64 (56%) RSV infections were community-acquired. The epidemiological curve of respiratory infections is shown in Fig 1.

### Infection control measures

Standard infection control measures included isolation of patients who were tested positive for one of the respiratory viruses until two consecutive negative swabs were obtained. Cohort isolation of patients infected with the same virus was possible. Contact patients were isolated until a negative swab was obtained at the end of the estimated incubation period. Masks were to be worn in all patient rooms on the transplant unit, on the intermediate care ward as well as when in contact with infected patients. Rigorous hand hygiene was implemented.

During the period of intensified screening, infection control measures were intensified to isolation of all newly admitted patients as well as all patients who developed respiratory symptoms until a negative swab was obtained. The number of visitors allowed was reduced to two visitors per person and children under the age of twelve were no longer admitted to the wards. Masks were obligatory on all the wards.

Neither visitors nor medical or nursing staff were screened for presence of respiratory viruses. However, visitors with respiratory symptoms were not admissible to the wards and medical or nursing staff experiencing respiratory symptoms was discouraged from having direct patient contact.

### Clinical outcome

The mean age of the all infected patients was 57.1 years (range 19–84 years) with a male:female ratio of 1.9:1. The distribution of the underlying hematological disorders was similar to the overall patient population at the department of hematology. The majority of infected patients (n = 75/111) underwent stem cell transplantation (42 autologous, 48 allogeneic, 15 autologous and allogeneic); 13 patients were infected pre-engraftment, 62 post-engraftment. All but three patients were considered immunocompromised for one or more of the following reasons: uncontrolled hematological malignancy, chemotherapy, < 3 months after transplantation, prior steroid treatment, systemic immunosuppression, reduced CD4+ T-cell count, hypogammaglobulinemia. Details on clinical characteristics of infected patients are presented in Table 1.

**Table 1:** Basic characteristics of patients with respiratory virus infections in the study cohort.

	all n = 111^a^	influenza n = 40	parainfluenza n = 13	RSV n = 64
**median age (range)**	57.1 (19–84) years	57.6 (21–84) years	60.6 (41–74) years	56.8 (19–73) years
**ratio male:female**	1.9:1	2.6:1	3.3:1	1.7:1
**underlying disease**				
AML/MDS	27	12	2	15
ALL/LBL	11	4	0	7
NHL/HL	27	10	3	16
MM	35	10	6	21
other	11	4	2	5
**transplant status**				
autologous	42	10	5	29
allogeneic	48	15	6	32
auto/allo	15	4	3	9
**LRTI**	48	15	5	28
**fatal outcome**	13	5	1	7

^a^ 6 patients with coinfections (n = 5 influenza/RSV, n = 1 parainfluenza/RSV)

ALL: acute lymphatic leukemia; AML: acute myeloid leukemia; HL: Hodgkin’s lymphoma; LBL: lymphoblastic lymphoma; LRTI: lower respiratory tract infection; MM: multiple myeloma; MDS: myelodysplastic syndrome; NHL: non-Hodgkin lymphoma; RSV: respiratory syncytial virus

Sixty four patients had URTI, 47 patients showed signs of LRTI, and 15 patients developed severe LRTI as defined by ICU treatment or fatal outcome. Thirteen infected patients with pneumonia died, despite ICU admission and supportive ventilation. Five of the fatal cases were infected with influenza virus, one with parainfluenza virus and seven with RSV. Oral tamiflu or ribavirin was given as treatment to 60 patients, immunoglobuline preparations to 13 patients, palivizumab was not administered. There were no significant differences in regard to demographic and clinical characteristics between influenza, parainfluenza and RSV infected patients. Furthermore, we could not identify any association between a specific virus group and a more severe course of illness. Co-infections were detected in 30 patients. A total of 44 different co-detected pathogens could be identified (bacterial 27, viral 11, fungal 6), e.g. *Pseudomonas aeruginosa* bacteremia, cytomegalovirus reactivation, and *Aspergillus fumigatus* pneumonia. Risk factor assessment amongst all infected patients showed by univariate analysis for the endpoint LRTI uncontrolled hematological disease (OR 4.39 [95% CI 1.70;11.84], p = 0.001), presence of co-infections (OR 5.82 [95% CI 2.07;17.82, p = 0.0002), and prior steroid therapy (OR 3.32 [95% CI 1.14;11.13], p = 0.02) as significant risk factors; a trend was seen for severe leukopenia (i.e. leukocytes <1/nl) >10 days (OR 3.22 [95% CI 0.88;13.34], p = 0.07). In regard of the endpoint severe LRTI only presence of co-infections (OR 4.89 [95% CI 1.31;19.39], p = 0.008) was a significant risk factor; a trend was seen for uncontrolled hematological disease (OR 3.42 [95% CI 0.93;13.28], p = 0.06). By multivariate analysis no significant influence factors could be identified.

### Phylogenetic analysis and genotype distribution pattern

**Fig 2:**
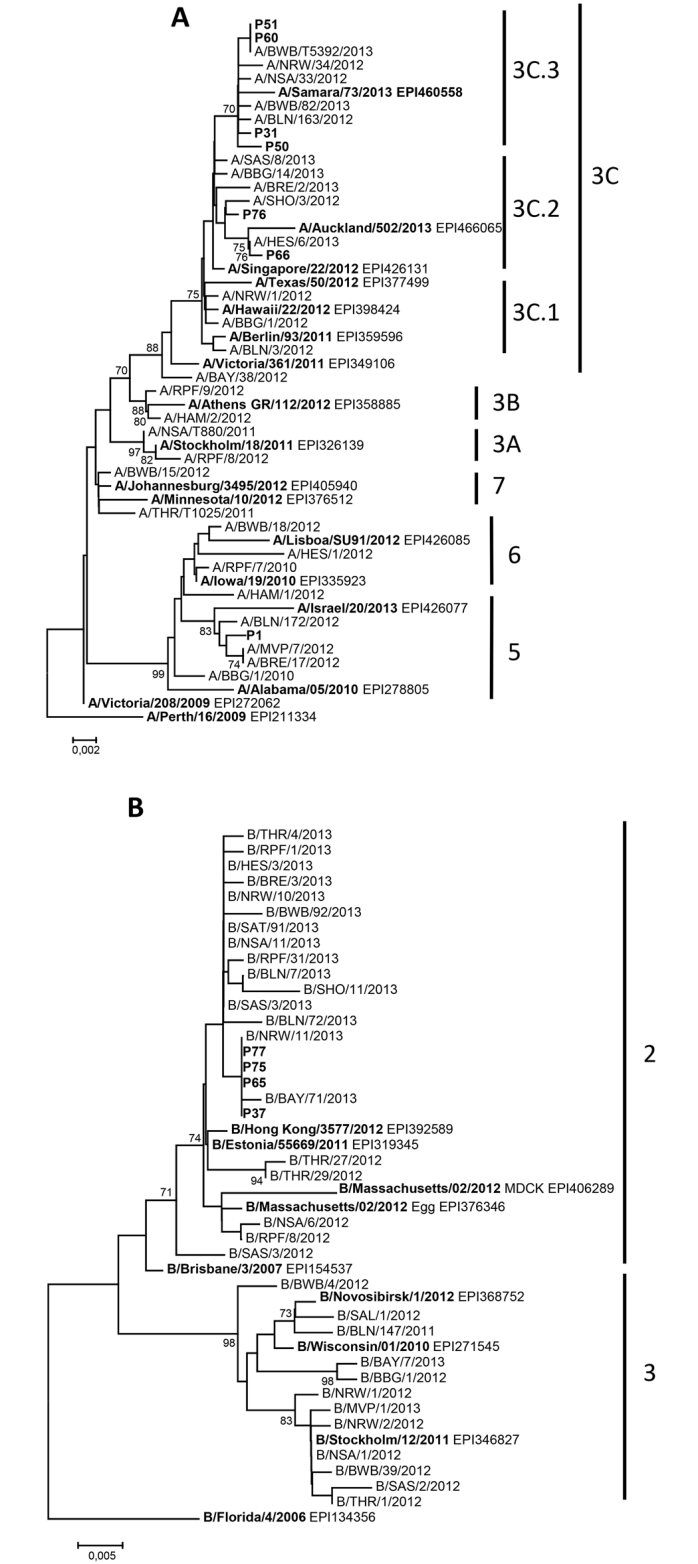
Phylogenetic tree of influenza A(H3N2) (A) (nt78-nt1061) and B/Yamagata-lineage (B) (nt100-nt571) virus sequences of the HA gene was constructed with MEGA version 5.2 using the neighbor joining method. Patient numbers are indicated, reference sequences for infuenza virus selected from GISAID are indicated by their accession numbers. Additionally, HA sequences from german national influenza sentinel are presented. The federal states are abbreviated as follows: Bayern (BAY), Baden-Württemberg (BWB), Berlin (BLN), Brandenburg (BBG), Bremen (BRE), Hamburg (HAM), Hesse (HES), Mecklenburg-Western Pomerania (MVP), Lower Saxony (NSA), North Rhine-Westphalia (NRW), Rhineland-Palatinate (RPF), Saarland (SAL), Saxony (SAS), Saxony-Anhalt (SAT), Schleswig-Holstein (SHO), Thuringia (THR). Bootstrap values greater than 70 are displayed on branch nodes.

**Fig 3:**
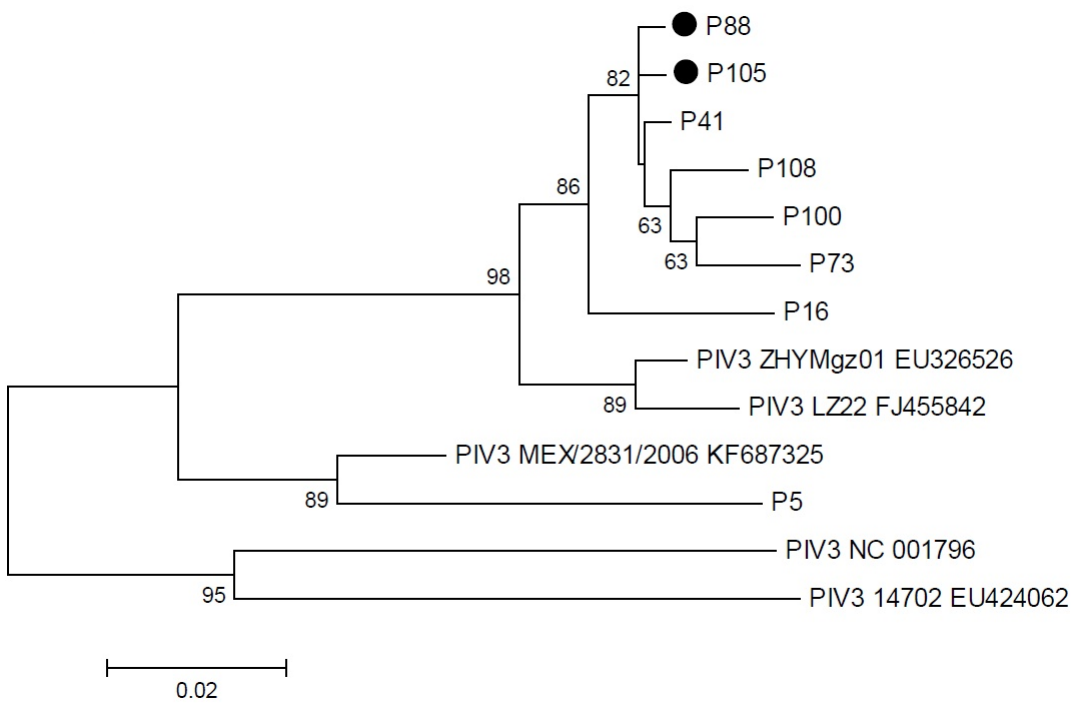
Phylogenetic tree of parainfluenza virus sequences from the fusion gene was constructed with MEGA version 5.05 using the maximum likelihood method. Patient numbers are indicated, reference sequences for parainfluenza virus type 3 selected from GenBank are indicated by their accession numbers. Bar indicates 0.1 nucleotide substitutions per site. Bootstrap values greater than 60 are displayed on branch nodes. Patient numbers of long-term virus shedding patients are indicated in bold.

For a subset of 15 influenza-infected patients, 10 parainfluenza-infected patients and all 64 RSV-infected patients, enough sample material was available for further phylogenetic analysis. For influenza virus, five samples were determined as influenza A(H1N1)pdm09, six samples as influenza A(H3N2) and four samples as influenza B viruses belonging to the Yamagata-lineage. Phylogenetic analysis of the A(H1N1)pdm09 HA sequences revealed that the HA genes form seven genetic groups, wherein the group 1 represents the reference strain A/California/7/2009. During the 2012/13 season group 6 and 7 viruses co-circulated in Germany. Group 6 viruses dominated comprising the genetic subgroups 6A, 6B and 6C. The here identified A(H1N1)pdm09 viruses belonged to the subgroups 6A (n = 1), 6C (n = 2), and group 7 (n = 2) (data not shown). Molecular analysis of the A(H3N2) viruses showed that two variants co-circulated in Germany during the season 2012/13 corresponding to the genetic groups 3C and 5. Group 3C viruses dominated comprising the genetic subgroups 3C.1, 3C.2 and 3C.3. The here detected A(H3N2) viruses belonged to group 5 (n = 1), subgroup 3C.2 (n = 2) and 3C.3 (n = 4). Among the identified 3C.3 viruses two HA sequences were identical (Fig 2A). Since the 2001/02 season, influenza B viruses of the Victoria- and Yamagata-lineage co-circulated with different seasonal prevalence in the northern hemisphere. In the season 2012/13 the Yamagata-lineage was more prevalent. These viruses are divided into group 2 and 3 viruses. All identified B/Yamagata-lineage viruses analysed here cluster in group 2 and were characterised by identical HA sequences (Fig 2B). Analyzed parainfluenza virus positive samples revealed type 2 (one sample), type 3 (8 samples) and type 4 (1 sample), no identical parainfluenza type 3 strains were found (Fig 3).

**Fig 4:**
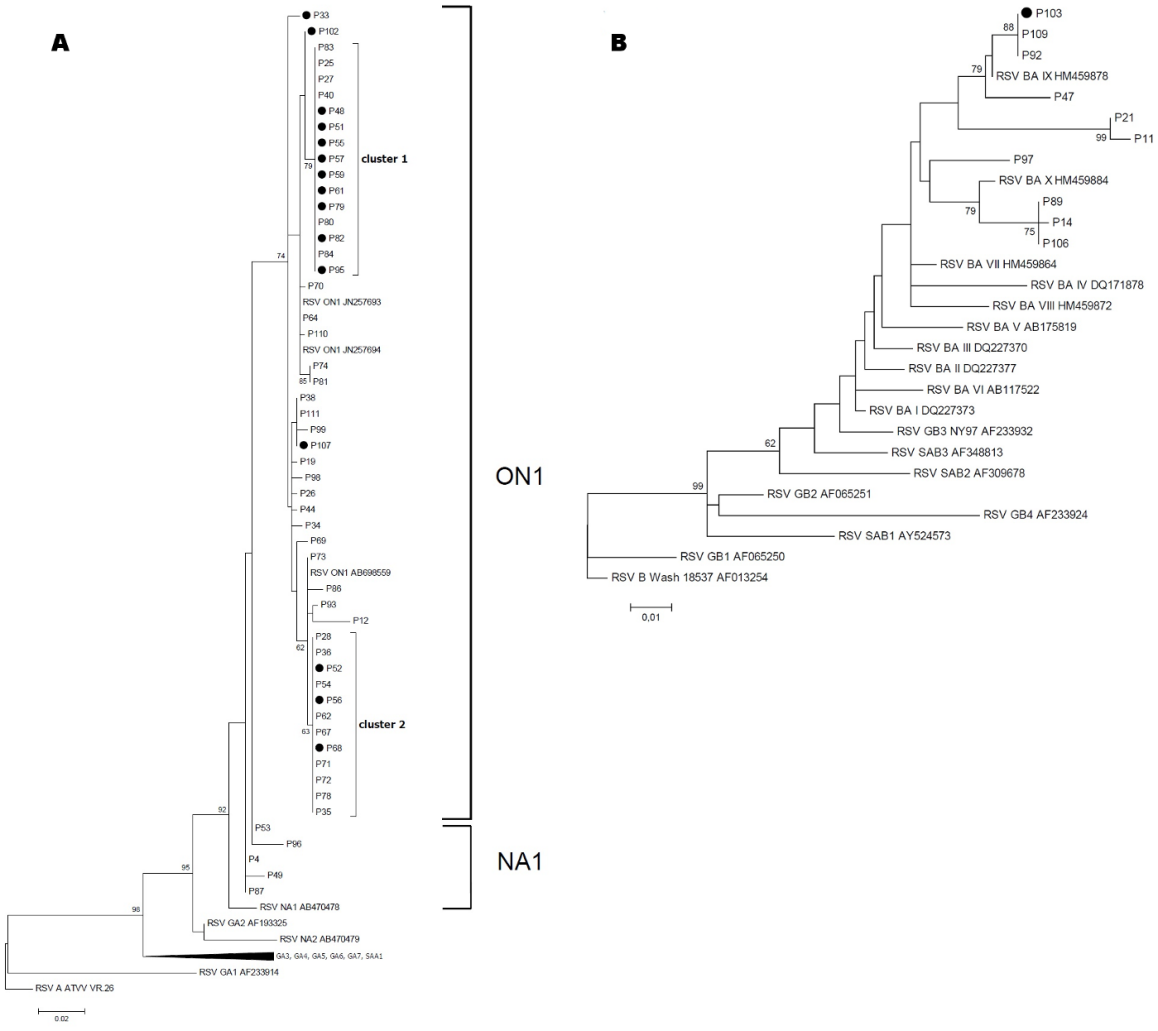
Phylogenetic trees of RSV A (A) and RSV B (B) virus sequences from the second hypervariable region of the attachment protein G gene were constructed with MEGA version 5.05 using the maximum likelihood method. Patient numbers are indicated, reference sequences for RSV A and RSV B selected from GenBank are indicated by their accession numbers. Bar indicates 0.1 nucleotide substitutions per site. Bootstrap values greater than 60 are displayed on branch nodes. Long-term RSV shedding patients are marked with a black dot.

RSV group specific PCR determined 54 RSV A and 10 RSV B infected patients. Sequences of the second hypervariable region of the G gene from these samples were successfully obtained and aligned with representative RSV sequences. Phylogenetic trees of RSV A and B sequences are shown in Fig 4A and 4B, respectively. The majority of RSV-A strains (n = 49, 90%) clustered with strains that were previously assigned to the novel ON1 genotype with a 72 nucleotide duplication, followed by strains clustering with genotype NA1 (Fig 3A). Two clusters were identified within the genotype ON1 subgroup, cluster 1 with 15 patients harboring identical RSV A ON1 strain G gene sequence and cluster 2 comprising 12 patients with another set of identical G gene sequence (Fig 4A). Thus, 27 (50%) of 54 RSV A infected patients carried similar virus strains. In each of these clusters, the first patient, i.e. P25 in cluster 1 and P28 in cluster 2 had a community-acquired infection, while all subsequently following patients were nosocomially infected. There were no significant differences in regard to severity and outcome of infection between the two clusters with 41% vs 42% asymptomatic/URTI only, 47% vs 33% non-severe LRTI, 12% vs 25% severe LRTI, and 12% vs 8% fatal outcome in cluster 1 and 2, respectively. All RSV-B strains (n = 10) clustered with strains that were previously assigned to the BA genotype with a 60 nucleotide duplication. BA strains could be further differentiated into genotypes BA IX and X (Fig 3B).

### Long-term virus detection

**Fig 5:**
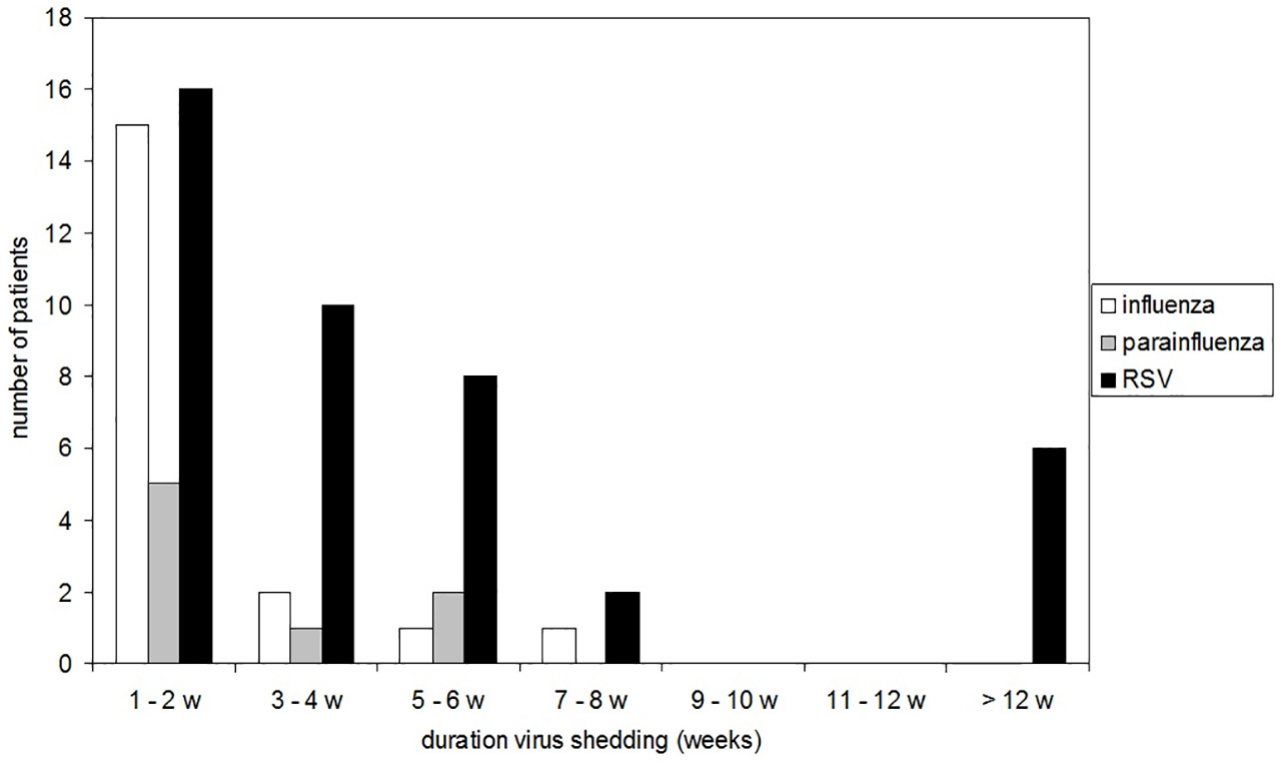
Duration of influenza virus, parainfluenza virus and RSV shedding periods.

Patients with respiratory virus infections were followed-up in order to analyze viral detection. Results for all infected patients with samples available from at least two consecutive weeks (n = 69) are shown in Fig 5. Long-term virus detectionfor more than 30 days was detected in 20 (29%) infected patients, i.e. two influenza virus infections, two parainfluenza virus infections, and 16 RSV infections (Table 2). Comparing clinical characteristics between patients with and without long-term virus detection revealed a significantly higher proportion of patients following allogeneic transplantation in the long-term virus detection cohort (14/20 vs 34/91 patients, p = 0.01). In this context it is worth noticing that 5 out of 6 patients with extreme long-term virus detection for ≥ 90 days had received an allogeneic transplant; the one patient without allogeneic transplantation (P52) had received an autologous transplantation but experienced graft-failure with long-term leukopenia. Regarding the morbidity associated with viral infection there was no significant difference in the rate of LRTI development between patients with and without long-term virus detection (11/20 vs 36/91, p = 0.22).

**Table 2:** Long-term viral shedding more than 30 days.

patient	sex	age	underlying disease	transplant status	URTI/LRTI	infection	virus type	shedding duration	clearance
P31	m	66	CLL	allogeneic	URTI	influenza	H3N2	48 d	yes
P32	m	34	ALL	-	URTI	influenza	H1N1	41 d	yes
P33	m	55	MM	auto/allo	LRTI	RSV	A ON1	35 d	yes
P48	m	52	MM	auto/allo	LRTI	RSV	A ON1 cluster 1	334 d	yes
P51	m	69	MDS	allogeneic	LRTI	RSV	A ON1 cluster 1	111 d	yes
P52	m	55	DLBCL	autologous	LRTI	RSV	A ON1 cluster 2	156 d	no^a^
P55	m	66	MM	autologous	URTI	RSV	A ON1 cluster 1	40 d	yes
P56	m	34	AML	allogeneic	LRTI	RSV	A ON1 cluster 2	90 d	yes
P57	m	53	MM	auto/allo	LRTI	RSV	A ON1 cluster 1	39 d	yes
P59	f	66	MM	auto/allo	URTI	RSV	A ON1 cluster 1	43 d	yes
P61	m	54	MM	autologous	URTI	RSV	A ON1 cluster 1	57 d	no^b^
P68	m	56	AML	-	LRTI	RSV	A ON1 cluster 2	33 d	yes
P79	f	38	ALL	allogeneic	URTI	RSV	A ON1 cluster 1	91 d	yes
P82	f	63	MM	auto/allo	LRTI	RSV	A ON1 cluster 1	38 d	yes
P88	m	62	AML	allogeneic	URTI	parainfluenza	3	30 d	yes
P95	m	51	pancr. cancer	allogeneic	LRTI	RSV	A ON1 cluster 1	91 d	yes
P102	m	49	ALL	allogeneic	URTI	RSV	A ON1	35 d	yes
P103	f	55	TNHL	-	LRTI	RSV	B BA IX	40 d	yes
P105	f	49	MM	auto/allo	LRTI	parainfluenza	3	36 d	yes
P107	f	71	Burkitt	auto/allo	URTI	RSV	A ON1	57 d	yes

^a^ died while still shedding,

^b^ lost to follow-up; m: male,

f: female, ALL: acute lymphatic leukemia; AML: acute myeloid leukemia; CLL: chronic lymphatic leukemia; DLBCL: diffuse large B-cell lymphoma; LRTI: lower respiratory tract infection; MM: multiple myeloma; MDS: myelodysplastic syndrome; RSV: respiratory syncytial virus; TNHL: T-cell non-Hodgkin lymphoma; URTI: upper respiratory tract infection

Six of 64 RSV-infected patients were infected for more than twelve weeks, all patients carried RSV A genotype ON1, and belonged to either ON1 cluster 1 or cluster 2. An extensively prolonged viral shedding was noted in this group of patients. One patient died while still being infected with RSV A ON1, one RSV A ON1 infected patient was lost to follow-up. However, 18 of 20 (90%) patients with prolonged infection cleared viral infection as demonstrated by negative follow-up samples (Table 2). Long-term RSV-infected patients presented with a median duration of viral shedding for 80 days, range 35–334 days (Table 2). Interestingly, 12 (75%) of 16 long-term RSV shedding patients were infected with identical RSV G gene sequences found in phylogenetic genotype ON1 clusters 1 and 2. Patient P48, diagnosed with multiple myeloma, had received autologous and allogeneic transplantation and was persistently infected for 334 days, followed by clearance of the infection.

## Discussion

This surveillance study of influenza virus, parainfluenza virus and RSV infections in a large cohort of patients with hematological malignancies provides insight into the molecular epidemiology and circulation patterns of subtypes and genotypes of respiratory viruses in immunocompromised adults combined with the prevalence, duration and clinical impact of prolonged viral shedding. Phylogenetic analysis revealed a variety of influenza A(H1N1)pdm09, A(H3N2) and influenza B/Yamagata-lineage, parainfluenza 3 and RSV A, B viruses; whereof about half of the patients (n = 54/111; 48.6%) were infected with RSV subtype A. Furthermore, we describe the predominance of the newly described RSV genotype ON1 strain in RSV-infected patients. Identification of two distinct clusters of RSV strains is consistent with nosocomial spread being responsible for a significant proportion of observed infections. A unique aspect of this analysis was the repeatedly performed screening of viral respiratory infections identifying 18% of patients as long-term infected with RSV with a median shedding duration of 80 days. The high prevalence of long-term shedding of respiratory viruses in immunocompromised patients might increase the risk of nosocomial spread of infections and should be taken into account when devising infection control measures.

In adult patients with hematological disorders and stem cell recipients, respiratory viruses are an important cause of life-threatening pneumonia, and are associated with substantial morbidity and mortality. A broader screening for respiratory pathogens in hematopoietic stem cell transplant (HSCT) recipients has been recommended by several expert groups [10, 11]. Nearly half of our parainfluenza infected patients developed LRTI, which is in accordance with a report of Nichols et al. [23]. Nine of the fatal cases underwent blood stem cell transplantation, three were autologous and six were allogeneic. Although respiratory viruses might cause fatal pneumonia, the contribution of these agents to the fatal outcome is difficult to assess. Recently, characteristic findings in chest CT scan at the beginning of RSV pneumonia with nodules and tree-in-bud sign often combined with bronchial wall thickening have been described as pathognomonic [24]. Systematic analysis of risk factors in this special cohort of patients revealed hypogammaglobulinemia to be a significant risk factor for RSV-associated morbidity and mortality, whereas treatment with oral ribavirin might provide a protective effect [18]. Progression of RSV infection from URTI to LRTI is associated with an increased risk of death in these patients, resulting in a 7%-70% case fatality rate among patients with hematological disorders [2, 25, 26]. In a study of Milano et al. [19, the morbidity and mortality were relatively low in HSCT patients infected with either rhinovirus or coronavirus, asymptomatic detections were common.

Molecular characterization of respiratory viruses has the potential to aid in the identification of infection chains. In our study cohort, nearly half of the patients with viral respiratory infections were infected with influenza virus or parainfluenza virus representing a broad spectrum of different strains and types. Similar results for infections with influenza and parainfluenza virus infections among patients with haematological disorders have been reported earlier [8]. Regarding the identified influenza infections in the immunocompromised patient group, A(H1N1)pdm09, A(H3N2) and influenza B viruses have been detected. A similar prevalence of co-circulating influenza virus types/subtypes during the season 2012/13 was reported by the national influenza surveillance system in Germany [27]. The phylogenetic analysis revealed that the prevalence of proven influenza A variants and influenza B-lineages in tested patient specimens matched with those reported from the national and European influenza surveillance systems [28]. Therefore, the same variants were identified in our population-based surveillance and within our study cohort and no difference between variants causing mild and severe clinical cases could be detected. Several nosocomial infections in immunocompromised patients have been described for A(H1N1)pdm09 [29, 30]. In the present study, the identical influenza A(H3N2) and influenza B/Yamagata-lineage HA sequences could give an indication of nosocomial transmission. Immunocompromised patients were characterized by longer viral shedding during the course of influenza virus infection [30]. The longer times of viral shedding in those patients might favour nosocomial infection chains. Several lineages of RSV within groups A and B co-circulate simultaneously in the population and their relative proportions may differ between epidemics, although group A viruses tend to predominate. Sequencing of the variable regions of the G protein gene has been widely used to further subdivide the two subtypes into genotypes and facilitated differentiation between RSV isolates. In 1998, Peret al. established a classification of different genotypes based on the analysis of the variability of RSV G gene [20]. To date, 11 RSV-A genotypes, GA1-GA5 [31], GA6-GA7 [32], SAA1 [33], NA1-NA2 [34], and ON1 [35], and 23 RSV-B genotypes, GB1-GB4 [31], SAB1-SAB3 [33], SAB4 [36], URU1-URU2 [37], BAI–BAVI [38], BAVII–BAX [39], BAXI [40], BAXII [41], and THB [42] have been established based on phylogenetic analysis of nucleotide sequences. Studies on RSV strains show an accumulation of amino acid changes over the years, suggesting antigenic drift-based immunity-mediated selection [32, 33]. The European pattern in seasonal activity of RSV usually alternates in a regular biennial rhythm; an early season with strong RSV activity is followed by a weaker late season [43]. A seasonality of viral acute respiratory infections influenced by meteorological conditions has been described previously [5]. The vast majority of identified RSV-A strains clustered with strains that were just recently assigned to the novel ON1 genotype with a 72 nucleotide duplication, first described by Eshaghi et al. [34] in Ontario, Canada, in 2010. In Germany, circulation of this genotype was reported for the first time in winter 2011/2012 [35]. In line with another study in Europe, this study reports ON1 as the predominant genotype during the RSV epidemic season 2012/2013, suggesting a rapid spread of this emerging RSV strain [36]. Two distinct phylogenetic clusters were identified within the genotype ON1 subgroup and fifty percent of RSV A infected patients carried similar virus strains. All RSV-B strains clustered with strains that were previously assigned to the BA genotype with a 60 nucleotide duplication [37]. RSV lineages similar to the strain in our patients were simultaneously circulating in Germany in the same winter in children [3]. In line with the gradual spread of the BA genotype, making it the dominant circulating RSV-B genotype today, might result in a shift to the predominant genotype ON1. There is an increasing number of reports from across the world describing this novel genotype and the following seasons will show its full impact on the evolution of RSV-A [39]. The nucleotide duplication of the ON1 genotype seems to be beneficial for the virus, the novel strain shows an improved ability to evade previously induced immunity in the population suggesting an immune-escape strategy. Several RSV outbreaks among hospitalized hematology patients have been reported in the past [17, 40]. In one study involving post bone marrow transplant patients, it was found that nosocomial infection with RSV occurred in almost half of all patients on the ward [8], nosocomial transmission might be reduced by an enhanced infection control program. The presence of two distinct clusters points to nosocomial transmission, detected one week or later after admission.

The mean incubation period of respiratory virus infections in immunocompetent patients is stated as four days, but can vary between two to eight days [42]. However, persistence of viral shedding has received little attention in immunocompromised patients. An important observation in this study was the prolonged viral shedding and hence the carrier status of the patients affected. Long-term viral shedding for more than 30 days was detected in 18% of patients in our patient cohort, mostly associated with RSV. Seventeen of 20 patients with prolonged viral shedding were transplant patients, 13 patients with allogeneic transplants. This finding is in line with a recent study from Heidelberg, where the majority of patients with respiratory viral shedding for more than four weeks were allogeneic transplant recipients [22]. According to Khanna et al. [43], duration of viral shedding is not associated with immunosuppression, RSV subtype or treatment regimen. Since these patients shed for a long time, it seems prudent to assume that these patients remain positive for the duration of their hospital stay. This finding confirms previous reports of prolonged shedding among immunocompromised patients [17, 19]. Shedding in hematology patients was reported for 2–4 weeks [43–45]. However, this is the first time that for RSV a median shedding duration as long as 12 weeks has been reported. Recently, Milano et al. [19] reported prolonged viral shedding in hematopoietic stem cell transplantation recipients. Median duration of viral shedding for rhinovirus and coronavirus was five weeks and four weeks, respectively, and prolonged shedding of more than three months was observed in some patients.

Nosocomial transmission seems to have been responsible for a significant proportion of the infections observed in our cohort of immunocompromised patients. One might speculate on a chain of infection consisting of a limited number of viral strains being introduced from the community to the hospital, both by the patients themselves as may be by visitors and staff, and spread then nosocomially, further facilitated by prolonged viral shedding in the immunocompromised host. To avoid this self-reinforcing circle early detection and isolation of infected patients as well as rigorous hygienic measures are of key importance. Infection control measures should include systematic screening of patients taking into account the incubation periods when testing contact patients, regular re-testing of positive patients to assess prolonged viral shedding, as well as—if feasible—preemptive isolation of possibly infected patients.

Influenza is preventable by vaccination, which is recommended for high-risk populations, immunosuppressed patients and patients with chronic medical conditions [46]. Vaccines for parainfluenza and RSV are not licensed yet, a newly developed vaccine against parainfluenza virus was immunogenic and well-tolerated in seronegative young children [6]. The development of a vaccine could prevent RSV-related pneumonia in adults [47] and induction of neutralizing antibodies with an RSV vaccine may potentially reduce disease severity in adult populations. New strategies such as RNAi therapeutics have been shown to exert potent antiviral effects against influenza, parainfluenza and RSV in vitro and in vivo [48]. Molecular epidemiological studies of these viruses provide useful information for the development of globally effective vaccines.

This study is subject to several limitations. The study population is a heterogeneous group in regard to a variety of aspects: patients and samples were obtained based on a routine screening for respiratory viruses. Initially, patients were screened for respiratory viruses due to clinically manifest respiratory symptoms, whereas later on screening was escalated to weekly screening of all patients regardless of clinical symptoms. In addition, a mixed cohort of in-patients and outpatients were included in this study. Regarding the treatment of viral infections, nearly all influenza-infected patients received oseltamivir, however therapies such as immunoglobulin preparations or ribavirin were optional for RSV infected patients and were given mainly at the discretion of the attending physician. Finally, both non-transplant and transplant patients were included. According to the ECIL guidelines [10], the panel of the tested viruses was restricted to influenza virus, parainfluenza virus and respiratory syncytial virus. Other viruses, e.g. metapneumovirus is not recommended in the ECIL first line testing of these patients and testing was not performed.

In conclusion, 17% of immunocompromised haematological patients who were screened for presence of influenza virus, parainfluenza virus or RSV were tested positive for one of these three respiratory viruses with RSV accounting for more than half of the detected infections the newly emerged RSV A genotype ON1 predominated in the study cohort. The global spread and progressive diversification of ON1 strains into several lineages resembles the evolution of the RSV-B BA genotype. Further molecular investigations are needed to understand possible immunological or biological causes of the improved viral fitness of genotypes containing a duplicated region in the G gene. In line with the emergence of the BA genotype, it can be hypothesized that genotype ON1 could spread in a similar way and several lineages might subdivide into further genotypes.

Distinct phylogenetic clusters of genotype RSV ON1 strains and identical HA sequences of influenza viruses identified in these patients might also indicate nosocomial transmission. Prolonged virus shedding was observed especially in patients with RSV infection and following allogeneic transplantation and might have facilitated nosocomial spreading. It poses a major challenge for infection control management in hospital settings with immunocompromised patients and should be taken into account when devising infection control measures.
